# Construction and Application of Farmers' Practical Teaching System in Vocational Education Based on Big Data Mining Technology

**DOI:** 10.1155/2022/6075719

**Published:** 2022-08-31

**Authors:** Wei Peng, Zhibin Tang

**Affiliations:** ^1^Institute of Higher Vocational Education, Yueyang Vocational and Technical College, Yueyang, Hunan 414000, China; ^2^College of Engineering and Design, Hunan Normal University, Changsha, Hunan 414000, China

## Abstract

With the establishment and perfection of social market economy, China has made changes to the disadvantages of farmers' vocational education system, such as singleness, backward educational means, and backward levels. Compared to traditional forms of farming, problems related to lack of farming expertise, poor scientific and technological awareness, and weak labor skills are analyzed by applying big data mining technology to retrieve key issues in order to establish a professional education system. Data mining can meet the needs of farming knowledge and rapidly develop into an automatic information farming model, which is an effective way to maximize and enhance professional knowledge. The establishment of a professional education system will train most scientific research members and further enhance diversified labor productivity. The experimental summary of this paper is as follows: (1) the relevant data need to be predicted before and after, the predicted experimental data will effectively improve students' grades, which is beneficial to the development of practical teaching, and the pretreatment stage plays a substantial role. (2) Among the three algorithms, the adaptive function of genetic clustering algorithm is obviously better than the other two algorithms, and the adaptive curve is relatively stable. (3) The comprehensive assessment of the course divides students into three categories: poor students, medium students, and excellent students, among which poor students account for 30%, medium students account for 50%, and excellent students account for 20%. (4) The standardization of the education system has brought users a good learning mechanism, in which the teaching resources have been strengthened, and users have very high satisfaction with the evaluation of the whole system.

## 1. Introduction

Farmers' vocational education system is established on the level of economic system, which adapts to the development of market economy and will improve agricultural development economy. Adapt to the basic conditions of taking rural areas as the primary industry, and extend the professional technology to ideological education. The Internet plays a key and substantive role, and will adopt diversified educational models to teach farmers about planting methods. Because the allocation of teaching time in the whole period needs to be detailed, it is necessary to avoid the confusion of relevant information, which is a kind of development and utilization of resources. Big data mining technology will retrieve and reserve the knowledge of farmers' professional education cognition, professional knowledge, technical training, and other personnel engaged in agricultural activities. School-running modes and means should be diversified, and school-enterprise cooperation, rural service, and other educational concepts serving the people should be implemented to run schools. This paper summarizes the innovation status of the pilot counties of new professional farmers and puts forward the theory of establishing and perfecting the policy system and giving full play to the role of the market [1]. In view of the confusion of the concept of new professional farmers, this paper re-examines the basic concept and connotation of the times of new professional farmers [2]. This paper expounds the connotation and realistic needs of new-type professional farmers and the cultivation experience of foreign professional farmers, and puts forward some supporting policies and measures for perfecting the cultivation of new-type professional farmers [3]. Strengthen the policy orientation of cultivating new professional farmers, and construct a new professional farmer cultivation system that embodies the concept of lifelong learning [4]. The meaning of professional farmers: New professional farmers have a broad and narrow distinction, and have a more conscious sense of responsibility and broader responsibility requirements [5]. On the issue of cultivating new professional farmers, we can learn from the practice of the USA and introduce practical laws and regulations [6]. Vigorously strengthen system construction, promote the integration of industrial chain, and strengthen the education and training of agricultural practitioners [7]. Construct flexible practical curriculum system, implement flexible talent training scheme, and explore practical teaching assessment methods with vocational education characteristics [8]. This paper discusses the characteristics of vocational education with multiple purposes and comprehensive contents, and cultivates new teaching forms [9]. A practical teaching system suitable for agriculture and forestry majors has been established, which enriches and expands students' practical skills [10]. Based on the analysis of China's modern agricultural development perspective, this paper puts forward some suggestions on how to build a platform and other aspects of the reform of the training mode of agriculture-related professionals in secondary vocational schools [11]. This paper expounds how the experimental teaching demonstration center can better serve the experimental teaching and the cultivation of innovative talents, and give full play to the radiation role of the experimental teaching demonstration center [12]. In-depth thinking on the curriculum of agriculture-related majors provides scientific ideas for the development of rural secondary vocational education [13]. To better cultivate students' practical ability and innovative and entrepreneurial ability, we should carry out practical teaching reform from the aspects of teaching mode and teaching team [14]. Taking individualized experimental teaching as the content, the practical teaching system of microbiology is constructed to meet the individualized needs of different individuals [15].

## 2. Theoretical Overview of Big Data Mining Technology

**Figure 1:**
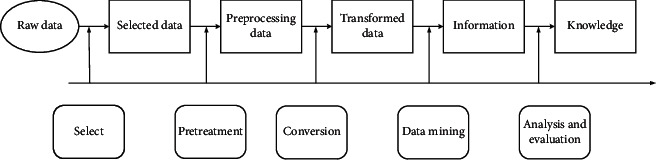
Basic process of data mining.

Big data mining technology extracts useful information from data sets and makes use of it, which is expressed in the form of rules, concepts, laws, and patterns. It can effectively help decision-makers to analyze the current situation, find hidden relationships, and then predict possible behaviors. The basic process is shown in Figure 1.

The whole process is a continuous feedback process. For example, if a bad extraction is found in the process, or the expected results are not achieved, it is necessary to start over.

There are two influencing factors in quality selection: first, whether the effectiveness of its products can be brought into play in this process, whether key factors can be extracted, and whether it can be applied in the actual situation; second, if there are errors or improper options, the conversion is unsuccessful, which will depend on the goodness of the extraction results.

### 2.1. A Survey of Clustering Algorithms

Clustering is an easy-to-understand way to gather related activities. Through the research and analysis of things, people can recognize related laws. There are also small classifications in clustering projects. Classification is to classify the regular characteristics known in advance, and other categories are the results of human analysis and research. In biology, cluster analysis can be used to deduce the related habits of plants and animals, analyze genes, and obtain inherent survival rules. Correlation calculation can be roughly divided into three categories: classification of fuzzy relation, fuzzy algorithm of objective function, and fuzzy algorithm based on neural network.

Partition clustering refers to dividing related combined data sets into *n* groups, which makes the objective function partition level smaller. Hierarchy refers to merging or splitting related passing objects until all conditions are met. Strictly implement the conditions of partition, people can grasp the key points when dealing with problems, and each can reflect the actual problems, thus becoming the mainstream research of cluster analysis and making use of it.

#### 2.1.1. K-Means Clustering Algorithm

K-means is the most classical clustering algorithm. Firstly, K objects are randomly selected, and each object represents a core. According to the distance between cores, similar type characteristics are assigned to one class for each subject. They are summarized as follows.

Nonlinear programming problem [16]: The classification algorithm is as follows:

(1) $$\begin{array}{c} minJ\left(W,P\right)=\overset{k}{\underset{i=1}{\sum }}\overset{n}{\underset{j=1}{\sum }}d^{2}\left(x_{j},p_{i}\right)\text{,}\\ s.t.\omega_{ij}\in \left\{0,1\right\},1\ll i\ll k,1\ll j\ll n\text{.} \end{array}$$

Among them,

(2) $$\begin{array}{c} W={\left[\omega_{ij}\right]}_{k\times n}\text{.} \end{array}$$

*W* is the partition matrix, which indicates that *J* samples belong to and do not belong to Class *I*, *P* is the cluster center, and *D* is the adaptation degree. The specific process is as follows:

  Input: The number of clusters contains *n* object data sets.
  Output: The value of the cluster.

The algorithm has good extensibility, and the difficulty of the algorithm can calculate the core quality results according to the number of clusters generated in advance. His shortcomings are that we must realize the results of understanding spherical clusters, but also consider the choice of initialization, so as to achieve the optimal processing. Secondly, the sensitive response of noise to the algorithm is very strong.

#### 2.1.2. Fuzzy C-Means Clustering

Fuzzy C-means (FCM) algorithm is an optimization algorithm based on K-means algorithm by introducing fuzzy matrix and dividing it. The objects considered in practice have not strict attributes, so they must be in the classification algorithm of fuzzy clustering. The algorithm is described as follows:

Mathematical programming problem [17] formula is as follows:

(3) $$\begin{array}{c} \min\;J\left(W,P\right)=\overset{k}{\underset{i=1}{\sum }}\overset{k}{\underset{i=1}{\sum }}{\left(\omega_{ij}\right)}^{m},d^{2}\left(x_{j},p_{i}\right),\\ s.t:\omega_{ij}=\epsilon \left[0,1\right],1\leq i\leq k,1\leq j\leq n\text{,}\\ \overset{k}{\underset{i=1}{\sum }}\omega_{ij}=1,1\leq j\leq n\text{,}\\ 0<\overset{k}{\underset{i=1}{\sum }}\omega_{ij}\leq n,1\leq j\leq n\text{,} \end{array}$$

where *m* represents the weighting index, and the algorithm is to find the fitness degree *d* of clustering center. Euclidean distance [18] formula is

(4) $$\begin{array}{c} d^{2}\left(x_{j},p_{i}\right)={\left(\overset{l}{\underset{s=1}{\sum }}x_{j}\left(s\right)-p_{i}{\left(s\right)}^{2}\right)}^{1/2}. \end{array}$$

In order to realize the clustering center P of the minimum membership matrix of the objective function *J* (W, P), it is necessary to cluster the fuzzy C of the mean value. The optimal solution of the objective function is the solution of the minimum value; that is, the center and membership degree are updated as follows:

(5) $$\begin{array}{c} \omega_{ij}=\frac{1}{\overset{k}{\underset{c=1}{\sum }}{\left(d_{ij}/d_{cj}\right)}^{2/\left(m-1\right)}}\text{,}\\ p_{i}=\frac{\overset{n}{\underset{j=1}{\sum }}\omega_{ij}^{m}x_{j}}{\overset{n}{\underset{j=1}{\sum }}\omega_{ij}^{m}\text{.}} \end{array}$$

**Figure 2:**
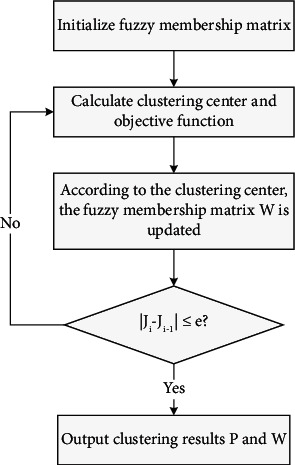
Basic flow of fuzzy C-means algorithm.

According to the above conditions, C-means can be modified repeatedly to achieve central clustering and membership matrix. The specific algorithm flow is as follows in Figure 2.

Fuzzy C-means algorithm is a popular algorithm nowadays, which is not only simple, but also fast, because it has a fairly perfect theoretical system; secondly, it can be applied in many wide fields as a simple practical tool. However, there are still some shortcomings, such as sensitivity to initial points and local optimal clustering results, and improved points can be divided into any point and any class of different degrees of clustering.

### 2.2. Genetic Algorithm

Genetic algorithm is a search method with self-adaptation and organization ability, which draws lessons from the principles of natural evolution and development of biology. Its principle is that each individual has its corresponding fitness value, which can be used to measure the quality of an individual. By imitating the principle of survival of the fittest of animals and plants in nature, the classification is carried out according to the fitness value. Individual crossover operation is to form new individuals through the combination of individuals, so as to form excellent individuals, which is similar to the process of gene selection.

The general working steps are as follows:

1. The input parameter set is transformed into bit string structure.
2. Defining fitness function.
3. Confirming genetic strategy.
4. Calculating fitness value.
5. Optimize the operation to generate the next stage group.
6. Output decoding and return to optimization operation again; in order to understand the unique deterministic index of individual survival chance selection of population, its fitness value is non-negative, and the larger the result, the better. For a given optimization problem, the evaluation of the objective function is also non-negative, and the transformed coordinate direction is a positive correlation direction. This shows that the encoding of the initial population has a direction, different individual spaces have their own patterns, and its theory is rotation and gambling selection.

#### 2.2.1. Genetic Clustering Algorithm

Genetic clustering algorithm divides all data elements corresponding to sample codes into a category, and this function is defined as Euclidean distance, which can be randomly generated and obtained. Genetic operation can find the global optimal solution without the influence of outliers, which requires large data, more classifications, faster convergence, and stronger practicability. Its function expression is as follows:

Adaptation function [19] is

(6) $$\begin{array}{c} \min\;J\left(W,P\right)=\overset{k}{\underset{i=1}{\sum }}\overset{k}{\underset{i=1}{\sum }}{\left(\omega_{ij}\right)}^{m},\\ s.t:\omega_{ij}=\epsilon \left[0,1\right]\text{,}\\ \overset{k}{\underset{i=1}{\sum }}\omega_{ij}=1\text{,}\\ 0<\overset{k}{\underset{i=1}{\sum }}\omega_{ij}\leq n\text{,} \end{array}$$

Through the definition of objective function, the minimum value is transformed into the maximum value. That is, the formula is

(7) $$\begin{array}{c} f=\frac{1}{J\left(W,P\right)}\text{.} \end{array}$$

It needs to expand the coding form, and real coding is to directly define animal and plant genes through parameter problems. The clustering problem directly defines the center code.

Clustering center [20] expression is

(8) $$\begin{array}{c} P={\left(p_{1},p_{2},...,p_{k}\right)}^{T}\text{.} \end{array}$$

*P* represents a one-dimensional vector.

Gene string [21] expression is

(9) $$\begin{array}{c} I=\left\{p_{11},p_{12},...,p_{1m},...,p_{k1},p_{k2},...,p_{km}\right\}\text{.} \end{array}$$

Next, the genetic strategy is implemented, which includes selection operator, crossover operator, and mutation operator. The Group *X* for which the size is defined is

(10) $$\begin{array}{c} X=\left\{x_{1},x_{2},...,x_{n}\right\}\text{.} \end{array}$$

Selection probability [22] is

(11) $$\begin{array}{c} p_{s}=\frac{f\left(x_{i}\right)}{\overset{n}{\underset{i=1}{\sum }}f\left(x_{i}\right)},i=1,2,...,n\text{.} \end{array}$$

Individual cumulative probability [23] is

(12) $$\begin{array}{c} p_{s}\left(x_{i}\right)=\overset{i}{\underset{j=1}{\sum }}p_{s}\left(x_{j}\right),i=1,2,...,n,j=1,2,...,i\text{.} \end{array}$$

When selecting, the interval is [0, 1], which obeys uniform distribution. When the individual xi with cumulative probability *p*_*s*_ is selected to enter the classification center, the judgment result of random number rand is as follows:

(13) $$\begin{array}{c} rand\leq p_{s}\left(x_{i}\right). \end{array}$$

This means that the operator is selected. At present, the crossover operator has the mating forms of one-point crossover, uniform crossover, and multi-point crossover. When it obeys the uniform distribution, it can choose a crossover position, and the expression result is as follows:

(14) $$\begin{array}{c} a\in \left\{1,2,...,m-1\right\}\text{.} \end{array}$$

The exchange of gene strings at this position is as follows:

(15) $$\begin{array}{c} x_{1}=\left\{x_{11},x_{12},...x_{1a}x_{2\left(a+1\right)},...x_{2\left(m-1\right)}x_{2m}\right\}\text{,}\\ x_{2}=\left\{x_{21},x_{22},...x_{2a}x_{1\left(a+1\right)},...x_{1\left(m-1\right)}x_{1m}\right\}\text{,} \end{array}$$

In this way, the information exchange process between genes is completed, and new gene combinations are produced.

The validity function of clustering: the main requirement of analysis is that the similarity of categories is higher and there are more categories, so it can be measured from the compactness and separation of clustering.

Compactness definition [24] is

(16) $$\begin{array}{c} comp=\frac{1}{n}{\overset{k}{\underset{i=1}{\sum }}\overset{n}{\underset{j=1}{\sum }}\left(\omega_{ij}\right)}^{2}\cdot d^{2}\left(x_{j},p_{i}\right)\text{.} \end{array}$$

Definition of separability [25] is

(17) $$\begin{array}{c} sep={\left(d_{\min}\right)}^{2}=\min\;{\left\|p_{i}-p_{j}\right\|}^{2}\text{.} \end{array}$$

The validity of clustering is directly proportional to compactness and conversely inversely proportional to separability. The greater the compactness value, the higher the classification similarity and the better the effect. On the contrary, the smaller the separation value, the better the clustering effect. Therefore, the validity function is defined as

(18) $$\begin{array}{c} S=\frac{comp}{sep}=\frac{{\overset{k}{\underset{i=1}{\sum }}\overset{n}{\underset{j=1}{\sum }}\left(\omega_{ij}\right)}^{2}\cdot d^{2}\left(x_{j},p_{i}\right)}{n\;\min\;{\left\|p_{i}-p_{j}\right\|}^{2}}\text{.} \end{array}$$

As a result, it is obvious that the maximized effectiveness *S* corresponds to the minimized comp, and the summary is the minimized objective function *J*. The requirement of analysis effectiveness is that the interior of classification is as similar as possible, and its exterior needs differentiation, which is to realize effective analysis of results by measuring the size of index.

## 3. Application of Algorithm Feature Module

**Figure 3:**
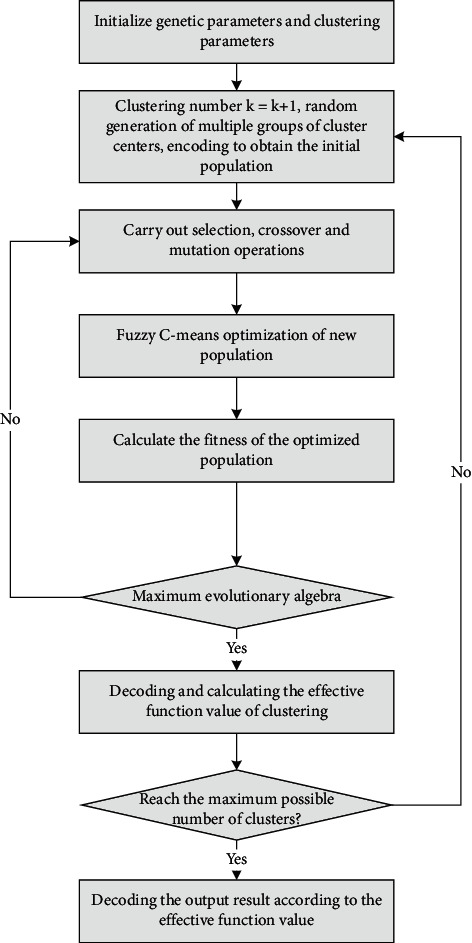
Flowchart of adaptive fuzzy C-means clustering algorithm.

Through the previous algorithm analysis, according to the strategy of genetic algorithm: selection, crossover, mutation, and a series of operations to produce new individuals, and then use fuzzy C-means for local optimization to speed up the algorithm. Its basic idea is as follows: firstly, encoding is done from randomly generated classification centers, and secondly, judging the termination condition: whether the maximum evolutionary algebra is reached. Otherwise, it is necessary to continue iteration, so that the maximum number of possible clusters can be judged, and then the transformation can be carried out. The application steps of adaptation are as follows in Figure 3.

The goal of feature analysis module is to group individuals into each group of features. Using the adaptive algorithm of this module to do system analysis can effectively reduce the irrationality of the initial cluster number, realize the evaluation and classification of individual overall features, and optimize the global parallelism of the system.

### 3.1. Data Collection and Preprocessing

#### 3.1.1. Collect Data

Collect relevant useful data according to the experimental requirements, including the original historical data. For example, the curriculum content, basic information, education system, and teaching tasks included in this experiment are all original data.

#### 3.1.2. Data Preprocessing

1. Remove irrelevant items
2. If the basic features of the original data have no intuitive effect on this experimental analysis, they can be removed.
3. Merging similar items
4. The practice of vocational education has relatively more performance assessment, which can be replaced by average performance in this system. There are still more practical training and theoretical study that can be comprehensively considered and merged.
5. Numeralization
6. Convert the hierarchy of excellent, good, medium, and poor into 90, 75, 60, and 50 of the percentile system.
7. Standardization
8. Through the unified processing in the preprocessing stage, the standard required by the data is adopted. If the corresponding table is used to express the students' course scores, the conclusions that are beneficial to the strategy analysis will be output.

### 3.2. Setting of Algorithm Parameters

Aiming at the problem of constructing the practical teaching system of vocational education in this paper, the clustering method is used to summarize and analyze, and the adaptive algorithm, fuzzy C-means algorithm, and genetic learning algorithm are provided. In order to reduce the workload and loss of classification and achieve global optimization, setting parameters have great influence on operation efficiency, which must be carefully considered. The setting parameters are as follows:

1. The maximum possible classification of data sets should not be less than 2 groups, and the best group number should be found between 2 and parameters.
2. The improvement of error accuracy is 0.01 by default.
3. Reducing the number of cycles of the algorithm and properly increasing the accuracy of parameters are helpful to improve the excellent classification.
4. Control the fuzzy degree of clustering process, too much classification is poor, and too little algorithm degenerates.

After setting the parameters, the system will automatically display the classification results and output relevant useful information. Set the unique parameters of genetic algorithm.

### 3.3. Clustering Analysis and Data Output

After clustering and parameters are set, you can view the proportion of various samples, the total scheme and variance of samples, the mean value of various types, standardization, and experimental results.

1. Sample ratio: Histogram, pie chart, and line chart can be used.
2. All kinds of variances of the total number of samples: histogram representation.
3. All kinds of mean values: rectangular coordinates represent abscissa courses and ordinate mean values.
4. The development of standardized curriculum implementation, expressed by curve.
5. Output the clustering results of each sample.

The output of effective function value avoids the oscillation phenomenon of genetic classification and analyzes the scores, preferred courses, and other related information of each educated person. On the one hand, the application of teaching system solves the arrangement of daily educational administration; on the other hand, it explores the effective use of clustering algorithm in student feature mining and gets good feedback.

## 4. Construction of Farmers' Practical Teaching System in Vocational Education

### 4.1. Comparative Analysis of Professional Farmers' Teaching Achievements before and after Data Preprocessing

**Figure 4:**
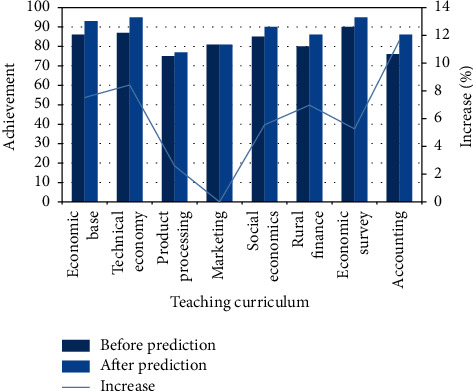
Comparative analysis of agricultural professional achievements before and after pretreatment.

According to the educational needs of farmers, the practical courses must be carried out, and the teaching tasks related to the needs must be conducive to the absorption of knowledge. Its technical economy, marketing, rural finance, etc., are all professional courses, and the efficiency and necessity of learning are analyzed before and after the prediction of achievement data sets. The results of the combination of theory and training are shown in Figure 4.

From the results in the figure, we can know that the results of relevant data preprocessing have been significantly improved, except for the course of marketing; accounting course is the largest increase of 12% after prediction processing, which shows that this course has increased by 10 points, which is the effective improvement of data quality and the importance of preprocessing.

#### 4.1.1. Output the Standardized Mean Value of Practical Courses

**Figure 5:**
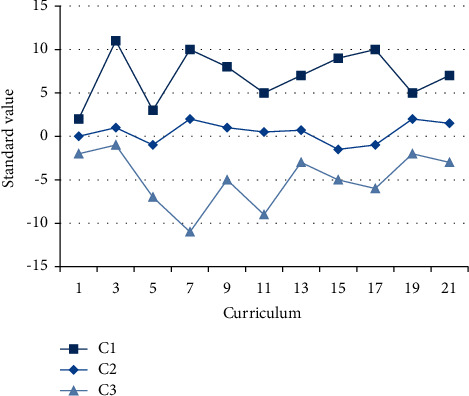
Average results of curriculum standardization.

After the end of the semester, the practical course is to judge the student's learning situation. The practical course may be agricultural experiment, the development of planting technology and sowing, which all reflect the significance of the course arrangement. The arrangement of teaching tasks is judged by the number of course sections. *C*1, *C*2, and *C*3 represent the three dimensions of the evaluation criteria, and the standard results under the dimensions are as follows in Figure 5.

It can be seen from the results in the figure that the standard values of dimension *C*1 are all greater than 0, the standard results of *C*2 fluctuate around 0, and the evaluation results of dimension *C*3 are all less than 0, indicating that there are obvious differences in the evaluation results under the three dimensions. When the evaluation system is realized, the accuracy of the evaluation index is increased, the complicated work is reduced, and the learning efficiency is improved.

### 4.2. The Value of Educational Curriculum Adaptation Function of Related Algorithms

**Figure 6:**
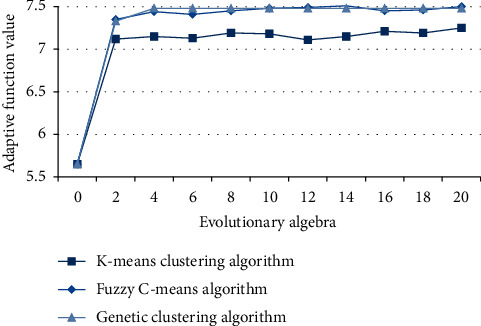
Evolutionary fitness curves of three clustering algorithms.

From the students' adaptation to the curriculum, we can get the degree of people's love for the curriculum and the rationality analysis of the number of school years. The previous three algorithms classify similar courses for better difference and plan the teaching arrangement for the next academic year from the students' adaptation. The experimental results are as follows in Figure 6.

From the numerical curve of fitness function, it can be clearly seen that genetic clustering algorithm is obviously superior to fuzzy C-means and K-means clustering algorithm, and has strong stationarity, while the other two kinds of algorithms have oscillation phenomenon. Genetic algorithm effectively avoids the fluctuation of average grades caused by the increase of the number of courses with the increase of evolutionary algebra, which will have a good preparation for teaching arrangement.

#### 4.2.1. All Kinds of Mean Curves after Teaching Standardization

**Figure 7:**
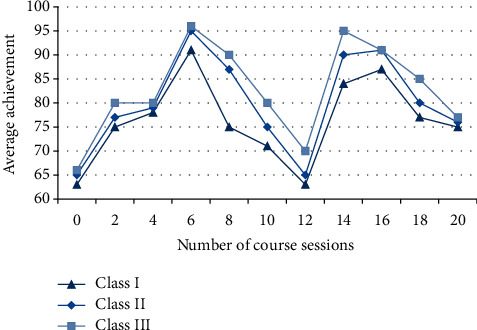
Average score curves of various algorithms.

After the standardization of the teaching system, the work will be carried out efficiently with this system, which greatly improves the teaching quality and reduces the time for students to adapt to teachers' teaching habits. The 20-session cycle course of the semester is a standardized time, which can complete the work tasks of the whole semester. The following results in Figure 7 are obtained by analyzing the scores of three types of students.

Class I belongs to the overall level of poor, accounting for 30% of the total number, and the results are not idealized; Class II belongs to the medium level of learning, which has a certain mastery of basic knowledge and needs to strengthen learning, accounting for 50% of the total number; Class III belongs to top students, with excellent grades, accurate mastery of knowledge and utilization, accounting for 20% of the total. Therefore, when carrying out professional skills training, we can strengthen innovative education and make technical ability develop in a balanced way.

### 4.3. Construction of Farmers' Practical Teaching System Based on Genetic Algorithm

**Figure 8:**
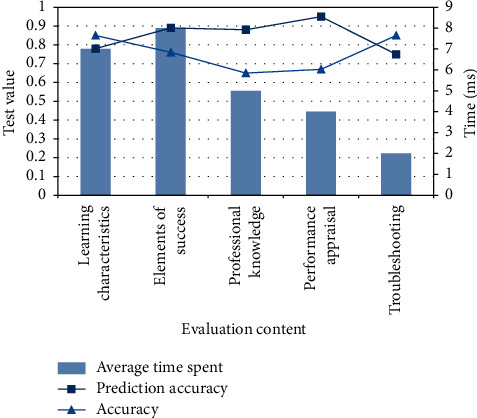
Experimental results of genetic algorithm.

Among the above three algorithms, genetic clustering algorithm is the best judgment for students to adapt to teaching, so according to the above results, it is determined to build a system with this algorithm. Teachers and students will have relevant key problems in the whole professional practice process, so this paper discusses the system evaluation. The experimental results are as follows in Figure 8.

It can be seen from the figure that the time spent on becoming a talent is the longest 8 ms in the whole test, because becoming a talent is a stage process that cannot be judged in a hurry, and difficult analysis is only a problem concept, so it takes relatively little time. The accuracy measurement under the whole algorithm mechanism is relatively stable without large fluctuation. However, the prediction accuracy needs to be improved.

### 4.4. Application of Vocational Teaching System

**Figure 9:**
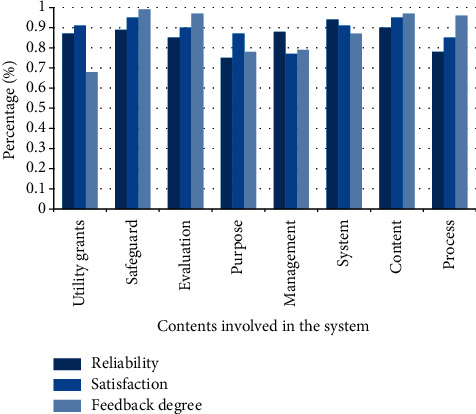
Questionnaire on the construction of teaching system.

Promoting the professional agricultural teaching system will provide farmers with a good way to know the blind spots; instead of blindly judging by previous planting experience, it now relies on standardized planting techniques. Enhance people's learning efficiency and train a large number of comprehensive talents. The survey results of user use are as follows in Figure 9.

Taking the algorithm as the theoretical basis of the whole system, through the investigation of users' reliability, satisfaction, and feedback, it is known that the feedback degree of users to the situation of UGC is the lowest 68%, which shows that the rich cultural heritage under the system lies in it. However, there are also problems that the security system has not been satisfied by users, and it needs to be optimized and improved.

## 5. Conclusion

Farmers' vocational education system is a complete system, which needs comprehensive analysis from all aspects and multi-angle consideration of the synergy of the whole system factors. In building a feasible education system, we should establish relevant infrastructure conditions to teach professional education for every educated person. This paper uses big data mining technology to construct the whole system, database retrieval, and the rapid construction of the whole educational administration system and applies it to educators. The dissemination of agricultural knowledge through professional education has made obvious changes in planting skills, increased income, and diversified planting. Big data mining technology has greatly improved the way of education, provided a solid theory for professional education reform, and effectively analyzed the reform system. The experimental conclusions of this paper are as follows: (1) for the introduction of educational curriculum information, fuzzy mean is introduced through clustering algorithm to optimize the processing, which overcomes the slow running problem of educational administration system. (2) The integration of peasant education in developing courses is studied, and data mining is used to analyze the influence of various educators' courses. (3) Explore the possible difficulties and conquering points in the whole learning process, so that people can understand knowledge more clearly. (4) Through comprehensive score prediction and analysis, the loopholes and optimization points in the education system are known, and the big data model is used as the basic theory to establish and optimize the system.

Experiments and Prospects: (1) The sparsity of data quantity and the authenticity of data will affect the accuracy of its results, and the quality of data sets should be greatly improved. (2) Because of the cultivation of professional knowledge in vocational education, more teachers and students resources should be mobilized to make use of. (3) With the continuous exploration of scientific research achievements, new teaching modes will inevitably appear, which is also the premise of promoting the reform of vocational education, and the continuous optimization of its system is also the basic work in the future. (4) To adapt to the specialized education mode, farmers should be regarded as the primary productive forces, but they should also learn modern mechanical work to adapt to the progress of the times.

## Data Availability

The experimental data used to support the findings of this study are available from the corresponding author upon request.
